# Supplementary figures and images for: Gut microbiome shifts with urbanization and potentially facilitates a zoonotic pathogen in a wading bird
Source: PLoS One. 2020 Mar 5;15(3):e0220926. doi: 10.1371/journal.pone.0220926 (PMC7058277; doi:10.1371/journal.pone.0220926)

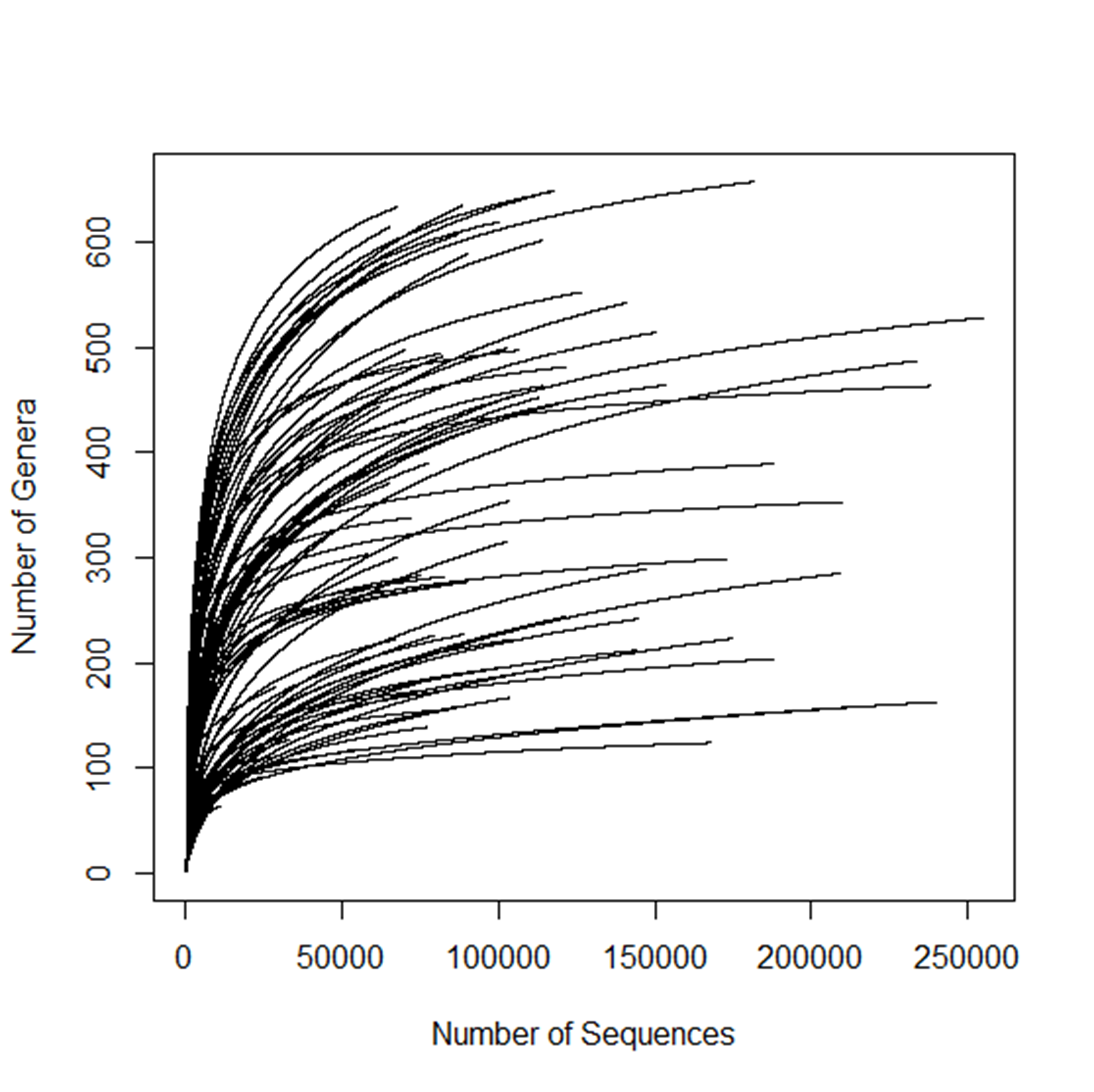

Supplement: S1 Fig — Rarefaction was performed using the rarecurve function in the vegan package [39]. (TIF) [file pone.0220926.s001.tif]
