# Supplementary material for: Gut microbiome shifts with urbanization and potentially facilitates a zoonotic pathogen in a wading bird
Source: PLoS One. 2020 Mar 5;15(3):e0220926. doi: 10.1371/journal.pone.0220926 (PMC7058277; doi:10.1371/journal.pone.0220926)
Supplement: S1 Table — (DOCX) [file pone.0220926.s002.docx]

Supporting Information S1 Table

|  |  | **Percent Surrounding Landcover** | | |
| --- | --- | --- | --- | --- |
|  | **Number of Samples** | **Wetland** | **Urban** | **Other*** |
| Wetland 2 | 1 | 91.8% | 0.0% | 8.2% |
| Wetland 7 | 3 | 91.8% | 0.0% | 8.2% |
| Wetland 5 | 3 | 74.4% | 15.4% | 10.2% |
| Wetland 6 | 3 | 73.8% | 4.3% | 21.9% |
| Wetland 1 | 5 | 26.8% | 65.5% | 7.7% |
| Wetland 3 | 2 | 15.2% | 25.5% | 59.3% |
| Landfill | 4 | 11.1% | 49.4% | 39.4% |
| Rehab Center 1 | 1 | 9.3% | 62.5% | 28.2% |
| Zoo 1 | 14 | 8.9% | 46.8% | 44.3% |
| Park 5 | 10 | 3.8% | 61.4% | 34.8% |
| Park 2 | 9 | 0.0% | 89.7% | 10.3% |
| Park 6 | 5 | 0.0% | 53.0% | 47.0% |
| Park 3 | 6 | 0.0% | 84.0% | 16.0% |
| Park 1 | 12 | 0.0% | 91.1% | 8.9% |
| Park 4 | 3 | 0.0% | 76.4% | 23.6% |
| Rehab Center 2 | 1 | - | - | - |

*Other land classifications included agricultural, coastal, other water, and other terrestrial
