# Supplementary material for: Gut microbiome shifts with urbanization and potentially facilitates a zoonotic pathogen in a wading bird
Source: PLoS One. 2020 Mar 5;15(3):e0220926. doi: 10.1371/journal.pone.0220926 (PMC7058277; doi:10.1371/journal.pone.0220926)
Supplement: S2 Table — (DOCX) [file pone.0220926.s003.docx]

Supporting Information S2 Table

|  |  |  | **Sequences** | |  | **Genera** | |
| --- | --- | --- | --- | --- | --- | --- | --- |
| **Site** | **Number of Samples** |  | **Mean Number** | **Standard Error** |  | **Mean Number** | **Standard Error** |
| Rehab Center 1 | 1 |  | 67605 | - |  | 633.0 | - |
| Rehab Center 2 | 1 |  | 63960 | - |  | 581.0 | - |
| Park 2 | 9 |  | 108408 | 15334 |  | 317.3 | 68.4 |
| Park 6 | 5 |  | 77400 | 18890 |  | 315.2 | 80.0 |
| Park 3 | 6 |  | 121111 | 21644 |  | 329.3 | 59.6 |
| Wetland 1 | 5 |  | 113303 | 32279 |  | 289.8 | 66.2 |
| Park 1 | 12 |  | 115061 | 18700 |  | 343.4 | 52.6 |
| Park 5 | 10 |  | 120359 | 11575 |  | 387.6 | 54.0 |
| Wetland 2 | 1 |  | 64762 | - |  | 370.0 | - |
| Wetland 3 | 2 |  | 175378 | 62761 |  | 457.5 | 5.5 |
| Park 4 | 3 |  | 74650 | 22398 |  | 351.3 | 137.4 |
| Zoo 1 | 14 |  | 102983 | 18523 |  | 302.3 | 42.0 |
| Wetland 5 | 3 |  | 89203 | 14894 |  | 410.0 | 119.1 |
| Wetland 6 | 3 |  | 131894 | 31528 |  | 420.0 | 61.2 |
| Landfill | 4 |  | 56060 | 17078 |  | 327.8 | 117.6 |
| Tetra Tech | 3 |  | 81505 | 194 |  | 465.0 | 27.5 |
| **All Samples** | **82** |  | **104695** | **6077** |  | **351.6** | **18.8** |
