# Supplementary material for: Gut microbiome shifts with urbanization and potentially facilitates a zoonotic pathogen in a wading bird
Source: PLoS One. 2020 Mar 5;15(3):e0220926. doi: 10.1371/journal.pone.0220926 (PMC7058277; doi:10.1371/journal.pone.0220926)
Supplement: S3 Table — (DOCX) [file pone.0220926.s004.docx]

Supporting Information S3 Table

| **Regression** | **Estimate** | **SEM** | **p-value** |
| --- | --- | --- | --- |
| Salmonella Prevalence~ |  |  |  |
| Mean Bacterial Diversity | -0.413 | 0.171 | 0.016 |
| Mean Bacterial Diversity~ |  |  |  |
| Bacterial Composition (PCoA1) | -0.392 | 0.149 | 0.009 |
| Percent Adult | 0.647 | 0.138 | <0.001 |
| Bacterial Composition (PCoA1)~ |  |  |  |
| Percent Urban Habitat | 1.180 | 0.209 | <0.001 |
| Mean Percent of Diet Provisioned | 1.514 | 0.694 | 0.029 |
| Mean Percent of Diet Provisioned~ |  |  |  |
| Percent Urban Habitat | 0.210 | 0.053 | <0.001 |

Model fit: Overall model fit p=0.223, Comparative fit index =0.950, Tucker-Lewis index=0.900
